# The Value of P‐Wave Parameters Changes in Predicting Catheter Ablation Outcomes for Paroxysmal Atrial Fibrillation

**DOI:** 10.1111/anec.70047

**Published:** 2024-12-31

**Authors:** Ibrahim Antoun, Xin Li, Zakariyya Vali, Ahmed Kotb, Ahmed Abdelrazik, Ivelin Koev, Riyaz Somani, G. André Ng

**Affiliations:** ^1^ Department of Cardiology, University Hospitals of Leicester NHS Trust Glenfield Hospital Leicester UK; ^2^ Department of Cardiovascular Sciences, Clinical Science Wing University of Leicester Leicester UK; ^3^ Department of Engineering University of Leicester Leicester UK; ^4^ National Institute for Health Research Leicester Research Biomedical Centre Leicester UK

**Keywords:** atrial fibrillation, catheter ablation, P‐wave, P‐wave amplitude, P‐wave duration

## Abstract

**Background:**

Pulmonary vein isolation (PVI) is the most promising management method for paroxysmal atrial fibrillation (PAF). The P wave in the electrocardiogram (ECG) represents atrial depolarization. This study aims to correlate P‐wave parameters after PVI with outcomes.

**Methods:**

This single‐center retrospective study included consecutive patients with first‐time PVI for PAF between 2018 and 2019 and targeted pulmonary veins (PVs). Procedure success was defined by freedom of ECG‐documented AF at 12 months. Digital 12 leads ECGs with 1–50 hertz bandpass filter were monitored before the procedure. P‐wave amplitude (PWA) and P‐wave terminal force in V1 (PTFV1) Corrected P‐wave duration (PWDc), and P‐wave dispersion (PWDisp), were measured before and after ablation.

**Results:**

The final analysis included 180 patients, of which 130 (72%) had successful ablations and 53 (30%) had radiofrequency ablation (RF). Males comprised 71% of the patients; the mean age was 60. Demographics were similar between both arms *p* < 0.001. Patients with failed PVI had increased PWDc after PVI (139–146 ms, *p* < 0.001) compared to patients with successful PVI. PWA increased significantly after failed PVI (1.6–2 mV, *p* < 0.001) and successful PVI (1.6–1.8 mV, *p* = 0.008). PWD (hazard ratio [HR]: 2.5, 95% confidence interval [CI]: 1.4–4.2, *p* < 0.001) and PWA (HR: 1.7, 95% CI: 1.2–2.9, *p* = 0.03) were independently associated with PVI failure at 12 months. PWdisp and PTFV1 were not correlated with outcomes.

**Conclusion:**

Increased PWDc and PWA after PVI were independently associated with failed ablation for PAF, supporting the role of P‐wave parameters in predicting outcomes.

## Graphical abstract

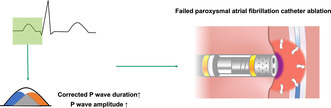

## 1 Introduction

Atrial fibrillation (AF) prevalence and incidence have grown over the last 20 years and will continue to increase over the following 30 years, becoming one of the largest public health challenges and epidemics globally (Lippi, Sanchis‐Gomar, and Cervellin 2021; Antoun et al. 2024a; Antoun et al. 2024b; Antoun, Aljabal et al. 2024). Pulmonary vein isolation (PVI) is a paroxysmal atrial fibrillation (PAF) rhythm control method (Cosedis Nielsen et al. 2012). This is mainly done by electrically isolating pulmonary veins (PVs). AF is proposed due to atrial cardiomyopathy (AC), a recently defined term encompassing changes in atrial structures and functions, including conduit, electrical conduction, reservoir, and contractile function (Goldberger et al. 2015; Goette et al. 2017). Evidence strongly suggests that AC, which is associated with a heightened risk of AF, is also linked to a greater incidence of AF‐related complications such as heart failure, cognitive decline, ischemic stroke, dementia, and mortality (Kamel et al. 2015). Notably, the associations of AC with neurocognitive and cardiovascular outcomes are independent of AF, stressing its prognostic importance. There is a need to discover methods that can accurately characterize AC and, importantly, be easily utilized in a clinical setting.

The 12‐lead ECG, a traditional clinical tool, is essential in detecting atrial cardiomyopathy. The normal P‐wave, generated by the atria, has multiple measured parameters, including morphology, duration, spatial axis, voltage, and area. Changes in these parameters could indicate atrial enlargement and conduction blocks which are known risk factors for AF and strokes. The predictive value of P‐wave parameters has been recognized for decades, with an advanced interatrial block being described in the 1980s as a marker for AF risk (Chen et al. 2022; de Luna, Oter, and Guindo 1989; Alexander et al. 2019).

Furthermore, P‐wave parameters, including (PWDisp) (Dilaveris and Gialafos 2001), P‐wave duration (PWD) (Nielsen et al. 2015), P‐wave dispersion, P‐wave amplitude (PWA) (Park et al. 2016), and P‐wave terminal force in V1 (PTFV1), (Morris Jr. et al. 1964; Gutierrez et al. 2019) have been associated with stroke, dementia, AF, and death. Other P‐wave measurements, including a notched P‐wave, duration‐to‐amplitude, and beat‐to‐beat variation, were correlated with PVI outcomes (Doğduş et al. 2022; Tachmatzidis et al. 2022; Okuyama, Kabutoya, and Kario 2024). Modifying the atrial substrate has been suggested to promote a change in P‐wave parameters. Previous studies compared P‐wave parameters after ablation with outcomes but only included certain parameters in certain leads (Salah et al. 2013; Miao et al. 2022; Ohguchi et al. 2021). Furthermore, PWD was not addressed in the heart rate in these studies. This study aimed to assess corrected P‐wave duration (PWDc), PWA, PWDisp, and PTFV1 in all 12‐leads ECG aiming to predict PVI outcomes for PAF.

## 2 Material and Methods

### 2.1 Study Design

This retrospective observational cohort study included consecutive patients > 18 years old who had their first PVI for PAF between January 2018 and December 2019 in Glenfield Hospital, Leicester, UK. Patients on amiodarone were excluded as amiodarone could alter P‐wave morphology (Burashnikov et al. 2008). Also, patients with atrial flutter, patients with previous PVI or flutter ablation, patients with a pacemaker, Patients with previous ablation procedures, patients with pacing devices, patients with moderate to severe valvular disease, patients with additional ablations outside PVs and patients who did not attend their follow‐ups were excluded. PVI was conducted by contact force RF, or second‐generation catheters were used for cryo. All involved patients had completed their PVI with confirmed bidirectional block. Failed PVI was defined by AF lasting for 30 s or more on Holter monitoring.

Patient demographics and medication details were obtained electronically by examining clinic appointment letters that provided clinical information, medications, ablation details, and follow‐up appointments. Three months after the procedure, all patients had a 7‐day ECG monitor to maintain sinus rhythm. Patients were followed up at 3, 6, and 12 months following the procedure with a 12‐lead ECG and a clinical review.

Patients who had first‐time PVI for PAF had continuous electronic. P‐wave parameters were measured before and after PVI and correlated with procedure success. PVI success was defined by the lack of ECG‐documented AF between 3 (blanking period) and 12 months following ablation using 12‐leads ECG or ambulatory monitoring. The study was reviewed and ethically approved by the University of Leicester Research Ethical Committee (REC) (reference number: 35479‐ia196). The study was reported according to the STROBE guidelines (Vandenbroucke et al. 2007).

### 2.2 Ablation Details

All procedures were performed as first‐time ablations in a single setting. In the radiofrequency (RF) procedures, a circular mapping catheter was deployed in the superior and inferior PVs. Circumferential ablation of the right‐sided and left‐sided ipsilateral PVs was conducted, guided by three‐dimensional left atrial (LA) mapping (CARTO3; Biosense Webster, California). A 3.5‐mm irrigated ablation catheter tip (ThermoCool SmartTouch Catheter; Biosense Webster) was utilized with ablation index (AI) guidance. Post‐procedure, dormant conduction in the PVs was examined using rapid adenosine triphosphate injection.

In the cryoballoon (cryo) ablation procedures, a long sheath (8.5 Fr SL0; Abbott Laboratories) was transitioned to a steerable sheath (FlexCath; Medtronic, Minneapolis, Minnesota), allowing for the insertion of a second‐generation cryoballoon (28 mm) into the LA over an inner‐lumen circumferential mapping catheter (Achieve, Medtronic). The cryoballoon was frozen at the ostium of the inferior and superior left and right PVs to achieve a bidirectional conduction block.

### 2.3 P‐Wave Analysis

The digital 12‐lead ECG has a resolution of 16 bits and a voltage range of 10 mV. The ECG data were filtered with a notch filter and a 1–50 hertz bandpass filter. One minute of ECG before and after PVI was exported from the system. P‐wave peak was detected with a minimum width/duration of 15 ms in the window of interest.

**FIGURE 1:**
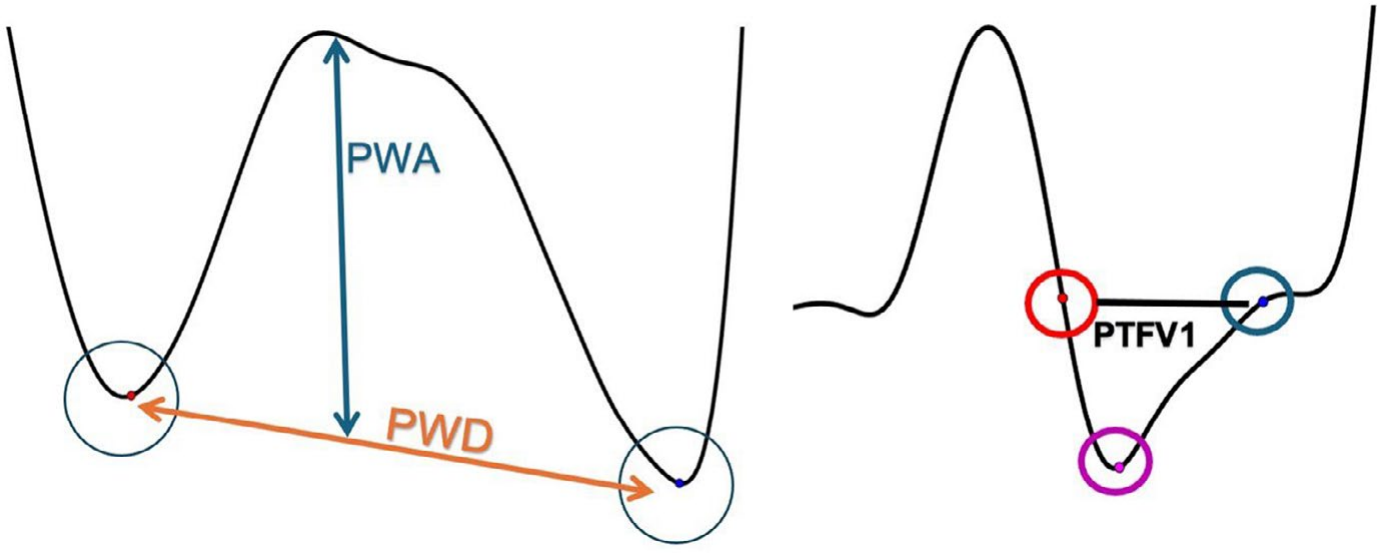
MatLab screenshots demonstrating P‐wave annotations. PTFV1, P‐wave terminal force in V1; PWA, P‐wave amplitude; PWD, P‐wave duration.

The P‐wave onset detection window was defined from the T‐wave end to the P‐wave peak. P‐wave onset was detected and determined by the point with a minimum perpendicular distance to the line connecting the P‐wave peak points and the two T‐waves (Figure 1). Twenty consecutive P‐wave measurements were averaged, and PWD was adjusted to the heart rate using the Hodges formula, similar to the QT interval adjustment. P‐wave beginning is defined by the first point of rise above the isoelectric line, while P‐wave peak is defined by P‐wave point with the most vertical distance from the isoelectric line. These could be manually adjusted during the measurement process.

The following P‐wave parameters are produced:

1. PWD: Distance from P‐wave onset to offset.
2. P‐wave amplitude (PWA): The distance from the isoelectric line to the P‐wave peak.
3. P‐wave dispersion (PWDisp): The max difference between PWDs.
4. P‐wave terminal force in V1 (PTFV1): The product of the maximum absolute amplitude and duration of the negative phase of the P wave in V1.

### 2.4 Statistical Analysis

Categorical variables were expressed as frequency and percentage. The mean and standard deviation were adopted to describe continuous parametric data. Pearson's χ^2^ or Fisher's exact test was used for categorical variables between groups. Paired, unpaired Student's *t*‐tests or Mann–Whitney‐*U* tests were used to compare continuous variables depending on the normality of the distribution. Cox hazards regression was conducted to assess independent predictors for PVI failure at 12 months. Variables that demonstrated significant findings in the univariable analysis were used in the multivariable analysis. A two‐sided *p*‐value < 0.05 was considered statistically significant. Statistical analyses were performed using GraphPad Prism V9.3 (San Diego, California, USA).

### 2.5 Study Outcomes

The co‐primary outcome included a correlation of PWDc, PWA, PWDisp, and PTFV1 with PVI outcome at 12 months.

### 2.6 Intraobserver Variability Test

An intraobserver variability test was conducted to assess the data's reproducibility. A randomly selected 22 ECGs were analyzed twice anonymously on two consecutive days across all 12 leads with a total of 5280 P‐waves. The highest variability was found in the PWDisp measurement (3.5 ± 0.3 ms, 16%), followed by PWA (0.02 mV ± 0.002, 11%), PTFV1 (0.3 ± 0.1 mm.s, 9%), and PWD (3.2 ± 0.2 ms, 3%).

## 3 Results

### 3.1 Patients Characteristics

**FIGURE 2:**
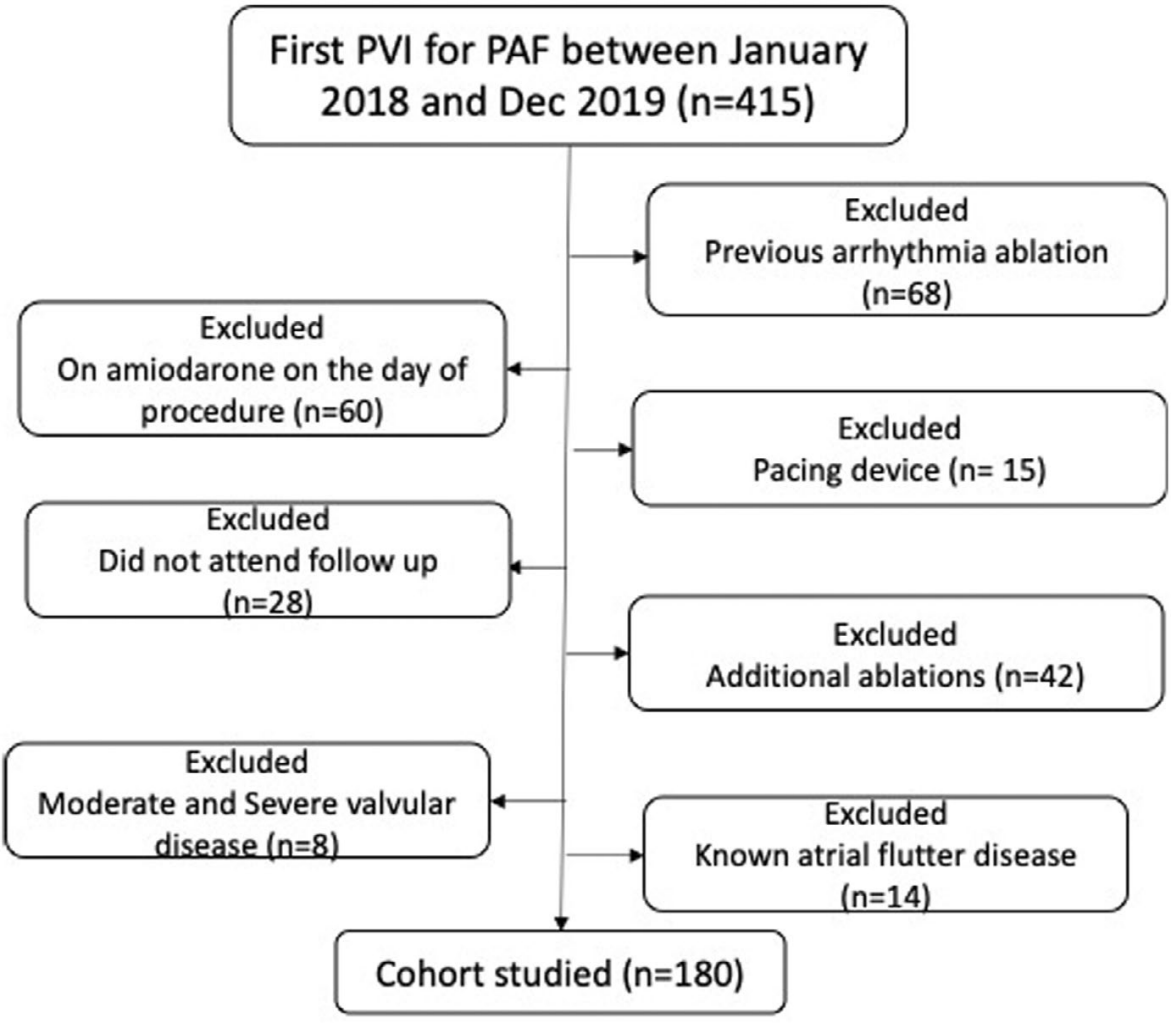
Flowchart demonstrating patient selection criteria. PAF, Paroxysmal atrial fibrillation; PVI, Pulmonary vein isolation.

After applying the inclusion and exclusion criteria in Figure 2, 180 PAF patients were involved in the final analysis. Of these, 130 (73%) had successful ablation at 12 months, and 53 (30%) had RF. Table 1 demonstrates demographics stratified by procedure success. Males comprised 71% of the patients, and the mean age was 60. There was no statistically significant difference in comorbidities between study arms. The proportion of patients with RF did not differ between successful and failed PVI.

**TABLE 1:** Demographics comparison between successful and failed first‐time ablation for paroxysmal atrial fibrillation.

	Total (180)	Success (*n* = 130)	Failure (*n* = 50)	*p*
Demographics				
Age (years)	60 ± 1.5	60.1 ± 0.9	60 ± 1.7	0.93
Male (%)	127 (71%)	94 (72%)	33 (66%)	0.47
Hypertension	64 (36%)	45 (35)	19 (38%)	0.67
Diabetes mellitus	20 (11%)	14 (11%)	6 (12%)	0.82
Congestive cardiac failure	12 (7%)	9 (7%)	3 (6%)	0.83
Ischemic heart disease	14 (8%)	11 (9%)	3 (6%)	0.58
Cerebrovascular event	15 (8%)	10 (8%)	5 (10%)	0.62
Body mass index (kg/m^2^)	22.2 ± 3.1	21.8 ± 4.8	23.1 ± 5.6	0.38
CHA_2_DS_2_Vasc score	2 ± 1	2 ± 0.7	2 + 1.3	0.87
Obstructive sleep apnea	12 (12%)	14 (11%)	7 (14%)	0.57
Active thyroid disease	27 (15%)	19 (15%)	8 (16%)	0.75
Blood results				
Potassium (mmol/L)	4.1 ± 1.8	4.3 ± 1.5	3.9 ± 1.6	0.65
Adjusted calcium (mmol/L)	2.3 ± 1.1	2.4 ± 1.4	2.3 ± 1.5	0.78
Magnesium (mmol/L)	0.9 ± 0.4	1.1 ± 0.3	0.8 ± 0.4	0.21
Heart structure				
Left ventricular ejection fraction (%)	51 ± 8	53 ± 9	48 ± 7	0.08
Indexed left atrial volume (ml/m^2^)	30.2 ± 0.6	31 ± 0.4	30.3 ± 0.8	0.71
Ablation technique				
Radiofrequency ablation	53 (30%)	42 (33%)	11 (22%)	0.2
Cryoballoon ablation	127 (70%)	88 (67%)	39 (78%)	0.11
Medications details				
Bisoprolol	164 (90%)	119 (92%)	45 (90%)	0.79
Verapamil	16 (10%)	11 (8%)	5 (10%)	0.71
Flecainide	49 (27%)	34 (26%)	15 (30%)	0.61
Sotalol	56 (31%)	35 (27%)	21 (40%)	0.12
Time AADs stopped after ablation (months)	6.3 ± 0.6	6.3 ± 0.4	6.3 ± 0.9	0.99

Abbreviation: AADs, antiarrhythmic drugs.

### 3.2 P‐Wave Parameters

**FIGURE 3:**
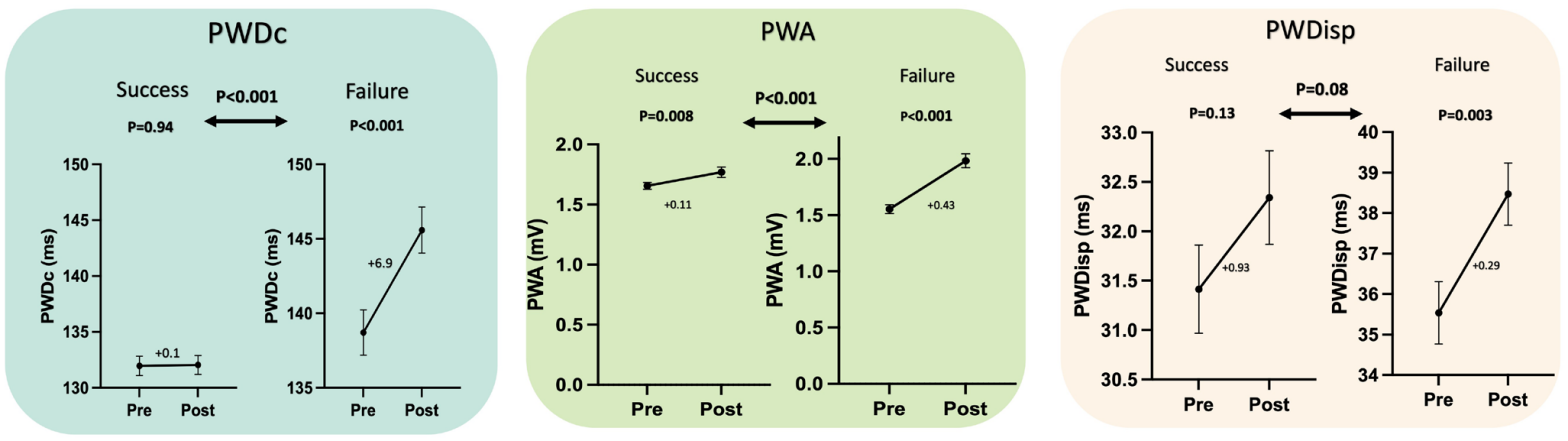
P‐wave parameters changes in successful and failed pulmonary vein isolation for paroxysmal atrial fibrillation. PWA, P‐wave amplitude; PWDc, Corrected P‐wave duration; PWDisp, P‐wave dispersion.

Figure 3 demonstrates the P‐wave parameter results between both arms. Patients with failed PVI had increased PWDc after PVI (139 ± 10–146 ± 9 ms, *p* < 0.001), compared to patients with successful PVI, in which PWDc remained unchanged from (132 ± 12 to 132 ± 11 *p* = 0.94). The difference in change was significant between both arms (*p* < 0.001).

Similar to PWDc, PWA increased significantly after failed PVI (1.6 ± 0.04–2 ± 0.1 mV, *p* < 0.001) and successful PVI (1.6 ± 0.03–1.8 ± 0.04 mV, *p* = 0.008). However, the increase in failed PVI was more significant than after failed PVI (+0.43 vs. +0.11 mV, *p* < 0.001). PWDisp was not changed significantly after failed PVI (36 ± 0.8–38 ± 0.8 ms, *p* = 0.07) and successful PVI (31 ± 0.5 ms–32 ± 0.5, *p* = 0.13). There was no significant difference between changes (*p* = 0.08).

PTFV1 was decreased significantly in successful PVI (−3.4–−4.6 mm.s, *p* < 0.0001) and failed PVI (−3.4–−5.4 mm.s, *p* < 0.001) without statistical difference between both arms (*p* = 0.18).

## 4 Discussion

This is the first study to correlate PWDc, PWA, PWDisp, and PTFV1 in all 12 leads after PVI with outcomes. Furthermore, correcting PWD to heart rate using the Hodges formula is a novel method we utilized in our previous work (Antoun, Li et al. 2024). The Hodges formula was chosen for its practicality and validated performance in electrophysiological interval correction (Chiladakis et al. 2010). Unlike nonlinear models, it provides a simple, linear adjustment that avoids overcorrection at extreme heart rates. This feature suits it particularly for retrospective studies with diverse patient populations, as seen in our cohort. This study provides valuable insights into the role of P‐wave parameters as predictors of PVI success in patients with PAF. Our findings demonstrate that increased PWDc and PWA are associated with favorable ablation outcomes at 12 months. These findings underscore the importance of detailed P‐wave analysis in the pre and postablation setting and suggest that certain ECG characteristics may be accessible and practical markers for ablation success.

### 4.1 Implications of PWDc and PWA Changes

The relationship between P‐wave parameters and PVI outcomes aligns with existing literature (Table 3), indicating that P‐wave features reflect underlying atrial pathology. Increased PWDc was more pronounced in patients with failed PVI in our study and has been shown to correspond with delays in atrial conduction (Ohguchi et al. 2021; Pranata, Yonas, and Vania 2019). The increase in PWDc after failed PVI may reflect residual or newly formed conduction abnormalities resulting from incomplete PVI. Persistent gaps or reconnections in ablated areas can prolong atrial conduction times, increasing PWDc. This phenomenon may not solely be due to pre‐existing substrate abnormalities. Still, it could also arise from iatrogenic effects of ablation, such as local tissue edema or fibrosis, which interfere with electrical propagation.

Increased PWA in successful and failed PVI cases was also notable, with a significantly larger increase among those with failed ablation outcomes. Previous studies have not described this novel finding (Table 2). The significant rise in PWA observed in patients with failed PVI could indicate compensatory electrical remodeling in response to incomplete ablation. Enhanced atrial wall tension or persistent electrical activity within the pulmonary vein‐left atrium junction may elevate atrial myocardial activation, manifesting as increased PWA. This finding aligns with prior studies linking PWA changes to atrial strain and compensatory hypertrophy (Andlauer et al. 2018), although the exact mechanisms remain under investigation. Importantly, our study evaluated PWA across all 12 ECG leads, providing a comprehensive view of atrial electrical activity, which may enhance the parameter's predictive accuracy. It is important to acknowledge that the immediate post‐ablation period is characterized by acute changes, such as inflammation and autonomic alterations, which may transiently influence P‐wave parameters. The observed increases in PWDc and PWA might partly represent these acute effects rather than solely reflecting the atrial substrate characteristics. Future studies incorporating longitudinal assessments beyond the immediate post‐procedural phase could help delineate these temporal effects.

**TABLE 2:** Cox regression to assess predictors for failed ablation at 12 months.

Variable	Univariable regression		Multivariable regression	
	HR (95% CI)	*p*	HR (95% CI)	*p*
PWDc (every 1 ms increase)	2.1 (1.5–3.9)	0.002	2.5 (1.4–4.2)	< 0.001
PWA (every 1 mV increase)	1.8 (1.2–3.2)	0.01	1.7 (1.2–2.9)	0.03
PWDisp (every 1 ms increase)	1.05 (0.9–1.2)	0.23		
PTFV1 (every 1 mm.s increase)	0.99 (0.8–1.2)	0.13		
Age (every 1‐year increase)	1.06 (0.6–1.6)	0.22		
Male (vs. female)	1.15 (0.7–1.2)	0.23		
Diabetes mellites (yes vs. no)	0.98 (0.8–0.1.1)	0.39		
Hypertension (yes vs. no)	0.98 (0.9–1)	0.36		
Congestive cardiac failure (yes vs. no)	1.16 (0.7–12)	0.46		
Cerebrovascular event (yes vs. no)	1.08 (0.8–1.3)	0.29		
CHA_2_DS_2_Vasc score (every unit increase)	0.95 (0.5–1.6)	0.15		
Active thyroid disease (yes vs. no)	1.05 (0.8–1.1)	0.39		
Obstructive sleep apnea (yes vs. no)	0.95 (0.7–1.3)	0.40		
Potassium (every 1 mmol/L increase)	0.9 (0.6–1.5)	0.33		
Calcium (every 1 mmol/L increase)	1.02 (0.8–1.3)	0.41		
Magnesium (every 1 mmol/L increase)	0.81 (0.4–1.5)	0.30		
Left ventricular ejection fraction (for every unit increase)	0.83 (0.5–1.7)	0.31		
Indexed left atrial volume (every 1 mL/m2 increase)	0.94 (0.2–2.1)	0.27		
Radiofrequency ablation (vs. cryoballoon ablation)	0.9 (0.5–1.4)	0.11		
Sotalol (vs. flecainide)	1.03 (0.7–1.4)	0.14		
Bisoprolol (vs. verapamil)	0.91 (0.4–1.6)	0.11		
Time antiarrhythmic stopped (every 1‐month increase)	0.86 (0.4–1.6)	0.36		

Abbreviations: CI, confidence interval; HR, hazard regression; PWA, P‐wave amplitude; PWDc, corrected P‐wave duration; PWDisp, P‐wave dispersion.

**TABLE 3:** Previous studies that correlated P wave changes after catheter ablation for atrial fibrillation with outcomes.

Author and year	N	AF	ECG	Recurrence	Cut‐off	*p*
Jiang et al. (2006)	108 (RF)	PAF	12 leads	↑ PWDisp		0.045
Ogawa et al. (2007)	27 (RF)	PAF (93%)	SAECG	↑ PWD		< 0.0001
Van Beeumen et al. (2010)	39 (RF)	PAF	12 leads	↑ PWD	≤ 5 ms change	0.04
Salah et al. (2013)	198 (RF)	PAF	Lead II	↑ PWDisp, ↓ PTFV1, ↑ PWD	PWDisp > 40 ms PTFV1 ≤ −0.04 mV.ms, PWD > 120 ms	< 0.001
Blanche et al. (2013)	102 (RF)	PAF (60%)	SAECG	↑ PWD	PWD > 140 ms	0.0008
Mugnai et al. (2016)	426 (RF)	PAF	12 leads	↑ PWDisp ↑ PWD		0.001
Hu et al. (2016)	171 (RF)	PAF	Lead II	↑ PWD		< 0.01
Nakatani et al. (2016)	126 (RF)	PAF (62%)	12 leads	↑ CVPD		< 0.001
Wu et al. (2016)	204 (RF)	PAF	12 leads	↑ PWD		0.006
Kanzaki et al. (2016)	76 (cryo)	PAF	SAECG	↑ PTFV1	> 9.3 mm.s	0.001
Knecht et al. (2018)	129 (RF)	PAF (65%)	12 leads	↑ PWD	> 120 ms	0.012
Yanagisawa et al. (2019)	115 (RF = 87)	PAF	12 leads	↓ Then ↑ PWD		0.001
Auricchio et al. (2021)	282 (RF = 30)	PAF (78%)	12 leads	↓ PWD	> 110 ms	0.012
Supanekar et al. (2021)	160	Nil	12 leads	PR ↑ and PWD ↓		0.03,0.02
Ohguchi et al. (2021)	84 (RF)	PAF (56%)	12 leads	↑ PWD	≥ 120 ms	< 0.001
Miao et al. (2022)	273 (RF)	PersAF	12 leads	↑ PWD		< 0.001

Abbreviations: AF, atrial fibrillation; Cryo, cryoballoon ablation; PAF, paroxysmal atrial fibrillation; PersAF, persistent atrial fibrillation; PTFV1, p‐wave terminal force in V1; PWD, P‐wave duration; PWDisp, P‐wave dispersion; RF, radiofrequency ablation; SAECG, signal average electrocardiogram.

### 4.2 Divergence of PWDisp and PTFV1 Findings With Prior Research

Contrary to prior studies, our findings did not demonstrate a significant correlation between P‐wave dispersion PWDisp and PVI outcomes. The previous literature showed that PWDisp increased at 3 and 6 months in successful PVI (Fujimoto et al. 2017), while another study demonstrated increased PWDisp in failed PVI without elaborating on measurement time (Mugnai et al. 2016). Our study measured P‐wave parameters directly after ablation, which might not match previous studies. The absence of a significant change in our cohort may suggest that PWDisp alone does not fully capture the complexity of conduction abnormalities in this patient population when measured directly after ablation. Our study's relatively homogenous baseline characteristics and stringent exclusion criteria (excluding patients with significant structural atrial disease or those on amiodarone) could account for this observation, as these criteria may limit the variability in PWDisp (Pérez‐Riera et al. 2016). Further investigation into specific patient subsets may be necessary to clarify the role of PWDisp as an indicator of PVI success. Furthermore, measuring PWDisp after 3 months of PVI may promote predictive findings.

The PTFV1 before ablation was not different between successful and failed PVIs. PTFV1 was described in 1964 (Kasser and Kennedy 1969) and correlated with the LA volume 5 years after (Morris Jr. et al. 1964). It represents the negative phase of the P wave in lead V1. It was considered abnormal when > 0.03 mm.s (Morris Jr. et al. 1964). Our study did not show a significant predictive value for PVI outcomes. Although PTFV1 has been linked to LA enlargement and structural atrial disease, which can affect AF management outcomes (Huang et al. 2020), the lack of association in our cohort suggests that PTFV1 may not be a universal predictor of procedural success. Notably, PTFV1 was reduced in both successful and unsuccessful PVI groups, indicating that changes in this parameter may not be specific enough to differentiate between outcomes. This finding suggests that while PTFV1 remains an important parameter for understanding atrial structure, its utility in predicting ablation success may be limited, particularly in populations with lower variability in baseline atrial characteristics.

### 4.3 Clinical Relevance and Application

The clinical utility of ECG‐derived P‐wave parameters in predicting PVI outcomes is significant, especially given the increasing incidence and prevalence of AF worldwide. Noninvasive markers such as PWDc and PWA are easily obtained and interpreted, allowing clinicians to assess patients' risk for PVI failure without invasive procedures. Identifying patients at higher risk for AF recurrence based on their P‐wave profile could inform preprocedural planning, aid in selecting alternative or adjunctive therapies, and potentially improve resource allocation in AF management (Chen et al. 2022). Moreover, these parameters may serve as useful endpoints in future trials exploring interventions targeting atrial remodeling or adjunctive therapies to enhance ablation efficacy.

Given the nuanced relationship between P‐wave characteristics and atrial remodeling, combining ECG parameters with advanced imaging techniques, such as magnetic resonance‐based atrial fibrosis assessment, may provide a more comprehensive evaluation of atrial substrate and AF risk (Daccarett et al. 2011). This integrative approach could enhance the prediction of PVI outcomes and inform patient‐specific management strategies.

### 4.4 Limitations

Several limitations should be noted. The study's retrospective nature limits causal inferences, and the single‐center design may limit generalizability. Additionally, while we excluded patients with potential confounders (amiodarone use, prior ablation, and structural abnormalities), these criteria may also limit the applicability of our findings to a broader AF population. The study did not utilize cardiac magnetic resonance (CMR) to evaluate LA fibrosis and is advised in future studies. The moderate sample size, particularly in the failed ablation group, could reduce the study's statistical power. Prospective, multicenter studies with larger and more diverse cohorts are needed to confirm these findings and explore additional ECG parameters and combinations thereof for predicting PVI outcomes. Although our sample size was relatively small, a post hoc calculation revealed a power of 81% to detect a difference in P‐wave parameters. While atrial substrate characteristics, such as fibrosis and conduction heterogeneity, are well‐established contributors to AF recurrence, our cohort's low incidence of structural abnormalities limits their explanatory power in this context. This underscores the need to consider procedural factors and post‐ablation dynamics alongside pre‐existing substrate features when interpreting P‐wave parameter changes. The study did not use long‐term rhythm monitoring, which might have missed asymptomatic AF. Finally, our study highlights the potential for integrating automated ECG analysis algorithms that incorporate artificial intelligence to assess P‐wave characteristics with greater precision and consistency. Machine learning algorithms could objectively assess parameters like PWDc and PWA and account for intraobserver variability, further refining the predictive power of ECG analysis in AF management (Nielsen et al. 2015; Park et al. 2016). While the Hodges formula offers significant advantages, it has limitations. It assumes a linear relationship between heart rate and conduction time, which may not capture nonlinear dynamics in certain patient subsets. Future work could explore alternative correction methods or validate the Hodges formula's application to PWD in diverse populations.

## 5 Conclusion

This study demonstrates that increased PWDc and PWA are independently associated with failed PVI at 12 months in patients with PAF, supporting the utility of these ECG‐derived parameters as noninvasive predictors of ablation outcomes. Our findings suggest that specific P‐wave characteristics may reflect underlying atrial substrate changes that influence the efficacy of PVI, providing valuable insight into personalized AF management. Further research is warranted to validate these parameters in diverse populations and explore their integration with advanced diagnostic tools for optimizing AF treatment strategies.

## Author Contributions

I.A. conceptualization, methodology, validation, and formal analysis were used to collect the data. X.L. validation, software, and data curation. A.K., Z.V., A.A., I.K., G.A.N., and R.S. writing – review and editing. G.A.N. supervision and writing – review and editing.

## Ethics Statement

The University of Leicester conducted and approved the project (reference number: 35479‐ia196), which involved prospective analysis of retrospectively collected anonymized data.

## Consent

The need for consent was waived.

## Conflicts of Interest

The authors declare no conflicts of interest.

## Data Availability

The data that support the findings of this study are available from the corresponding author upon reasonable request.
